# Neuropsychological Predictors of Trauma Centrality in OIF/OEF Veterans

**DOI:** 10.3389/fpsyg.2017.01120

**Published:** 2017-06-30

**Authors:** Roland P. Hart, Rohini Bagrodia, Nadia Rahman, Richard A. Bryant, Roseann Titcombe-Parekh, Charles R. Marmar, Adam D. Brown

**Affiliations:** ^1^Department of Psychiatry, School of Medicine, New York University, New YorkNY, United States; ^2^School of Psychology, University of New South Wales, KensingtonNSW, Australia; ^3^Department of Psychology, Sarah Lawrence College, BronxvilleNY, United States

**Keywords:** PTSD, military, centrality of events, working memory, cognitive flexibility, autobiographical memory

## Abstract

This study examined whether reduced performance on two neuropsychological tasks, cognitive flexibility and working memory, were associated with higher levels of trauma centrality. A growing body of research has shown that trauma centrality, the extent to which a person believes a potentially traumatic event has become central to their self-identity and life story, is associated with post-traumatic stress disorder (PTSD). Furthermore, PTSD is often associated with alterations in neuropsychological functioning. The relationship between neuropsychological processes and trauma centrality, however, has yet to be explored. OEF/OIF combat veterans (*N* = 41) completed the Post-traumatic Diagnostic Scale (PDS), the Beck Depression Inventory-II (BDI-II), the Centrality of Event Scale (CES), and on-line measures of cognitive flexibility and working memory assessed via WebNeuro. Bivariate Pearson correlations showed that CES scores were positively correlated with PDS and BDI scores, and negatively correlated with cognitive flexibility and working memory. Linear regressions revealed that working memory significantly predicted CES when controlling for depression and PTSD severity while cognitive flexibility approached significance when controlling for these same variables. This study employed a cross-sectional design, precluding causality. The small sample size, entirely male sample, and use of an online neuropsychological assessment warrant follow-up research. Although numerous studies have found an association between CES and PTSD, this is the first to suggest that neuropsychological processes underlie the construct of trauma centrality. Given the importance of maladaptive cognitive processes underlying the pathogenesis of PTSD, these data suggest that future studies aimed at examining the link between neuropsychological processes and maladaptive cognitive processes, such as trauma centrality, may help to characterize and treat PTSD.

## Introduction

The way in which a person remembers and interprets the impact of a traumatic event appears to play an important role in the pathogenesis of post-traumatic stress disorder (PTSD) (Horowitz, 1997; Foa et al., 1999; Conway and Pleydell-Pearce, 2000; Ehlers and Clark, 2000; Dalgleish et al., 2003; Bryant and Guthrie, 2007). In particular, a growing body of research has shown that PTSD symptoms are positively associated with *trauma centrality*, the extent to which a traumatic event is viewed as central to one’s identity and as a turning point in one’s life story (Berntsen and Rubin, 2006, 2007). Trauma centrality has been measured with the 20-item (or abridged seven-item) self-report measure, the Centrality of Event Scale (CES) (Berntsen and Rubin, 2006, 2007). Numerous studies have shown that higher scores on the CES are positively correlated with greater PTSD symptom severity in both clinical and non-clinical populations including college students (Berntsen and Rubin, 2006, 2007; Robinaugh and McNally, 2010), combat veterans (Brown et al., 2010), and adults with a history of childhood sexual abuse (Robinaugh and McNally, 2011). The relationship between trauma centrality and PTSD symptom severity has been shown to be significant even after controlling for stress, anxiety, depression, dissociation, negative perspective, and emotional intensity (Berntsen and Rubin, 2007; Schuettler and Boals, 2011). Most research examining trauma centrality has been cross-sectional, however, a recent prospective study found that trauma centrality longitudinally predicted PTSD symptoms (Boals and Ruggero, 2015). Although the relationship between trauma centrality and PTSD symptomatology is well-established, the extent to which neuropsychological processes underlie trauma centrality has yet to be examined.

Although studies have yet to test the association between neuropsychological performance and trauma centrality, prior theories and research suggest that alterations in cognitive and neuropsychological processes are intertwined with changes in self-related processes, such as recall for autobiographical memories. Neuropsychological impairments are related to the construction of maladaptive self-representations that have been proposed to underlie the onset and maintenance of PTSD. For example, there is now a robust body of research showing a link between PTSD and impairments in recalling specific autobiographical memories (e.g., Aupperle et al., 2012; Polak et al., 2012; Scott et al., 2015). Additionally, individuals with PTSD are more likely to recall trauma-related memories than trauma-exposed individuals without PTSD (for reviews see, Moore and Zoellner, 2007; Williams et al., 2007; Lapidow and Brown, 2015). These impairments in autobiographical memory may be linked with deficits in neuropsychological functioning, as overgeneralized memory in depression has been associated with impairments in working memory, verbal fluency, and executive functioning (for a review see, Sumner, 2012).

Neuropsychological research aimed at characterizing and studying healthy and clinical populations suggest that two executive functioning processes, working memory and cognitive flexibility, are associated with processes involved with the capacity to generate autobiographical memories and self-representations (e.g., Piolino et al., 2009; Prebble et al., 2013). Working memory, or the ability to process and manipulate information that is no longer perceptually present (Baddeley and Hitch, 1994; Smith, 1999), has been proposed to contribute to the “working self” in cognitive models of autobiographical memory (Conway and Pleydell-Pearce, 2000). The working self, or one’s hierarchy of active personal goals and self-image, has been suggested to serve as a schema, guiding the accessibility and content of autobiographical memories. Given that the working-self has been proposed to be a part of working memory (Conway, 2005), deficits in working memory may alter this top–down process and impair one’s ability to recall non-trauma related memories. Similarly, cognitive flexibility refers to the readiness to switch attentional focus between multiple concept systems depending on environmental stimuli; one’s ability to think about multiple concepts simultaneously or to change how one thinks about concepts (Scott, 1962; Diamond, 2013). As proposed by Ehlers and Clark (2000), people with persistent PTSD often have autobiographical disturbances that inhibit their ability to reorganize previous and future experiences, thus impeding an established stable view of themselves. Having a deficit in cognitive flexibility, then, may be related to one’s inability to think of themselves outside of the context of their trauma.

Theoretical frameworks have posited that maladaptive personal schemas for traumatic events are in part linked with neuropsychological impairments, such as deficits in working memory (Ehlers and Clark, 2000; Dalgleish, 2004). Specifically, working memory has been proposed to contribute to the ability to hold multiple self-representations and appraisals (Dalgleish, 2004). The Schematic, Propositional, Analog, and Associative Representational Systems (SPAARS) model (Power and Dalgleish, 1997, 1999; Dalgleish, 1999) proposes that working memory manipulates schematic (abstracted, generic knowledge), propositional (referential meaning in verbal form), and analogical (non-verbal referential information) formats of mental representation. SPAARS accounts for cognitive processing and representation, and explains that while the propositional and analogical levels of processing involve more basic manipulations of thoughts and mental images, schematic level representations are constructed and integrated *using* the propositional and analogical levels. In other words, analogical representations, such as sight and smell, and propositional representations, or thought content, of a particular autobiographical event combine to create a schematic sense of the entire experience greater than its component parts (Dalgleish, 2004). Discrepancies between schematic representations and active information, processed through working memory, are detectable in the SPAARS model, but a disruption of working memory could potentially leave schematic representations of oneself or an autobiographical event unchallenged. Thus, higher scores on the CES, which may reflect difficulty in generating non-trauma related self-representations, may be associated with a reduction in working memory.

The goal of the present study was to assess the relationship between trauma centrality, neuropsychological functioning, and PTSD symptomatology in a sample of US military Iraq and Afghanistan veterans. We hypothesized that neuropsychological performance, specifically working memory and cognitive flexibility, would be negatively associated with CES total scores. Similar to previous findings (Berntsen and Rubin, 2006, 2007), we hypothesized that CES total scores will be positively correlated to clinical measures of PTSD and depression. Moreover, we expected to find that the aforementioned neurocognitive measures would maintain their negative association with trauma centrality when controlling for PTSD symptom severity, thus linking deficits in working memory and cognitive flexibility directly to trauma centrality. Because of the known relationship between executive functioning and depression (see Veiel, 1997; Harvey et al., 2004), depression will be controlled for in analyses.

## Materials and Methods

### Participants

Operation Enduring Freedom (OEF) and Operation Iraqi Freedom (OIF) combat veterans (*N* = 41, *M_Age_ =* 33.02, *SD* = 6.31) with current PTSD (*n* = 11) and without current PTSD (*n* = 30) were recruited from the Mental Health Services of the Manhattan, Bronx and Brooklyn Veterans Affairs Medical Centers, other regional VA medical centers, Veterans Service Organizations, National Guard, reservist agencies and organizations, and from the general community. Recruitment methods included flyers, in-person presentations, media advertisements, internet postings (e.g., Craigslist), and referrals from clinicians. All study procedures were approved by NYU’s IRB and all participants provided written, informed consent. Participants were excluded if they had a lifetime history of psychosis, bipolar disorder, major depression with psychotic features, pre-deployment obsessive compulsive disorder, or were less than 2 months stable on psychiatric medications. Participants with exposure to trauma within the past month or with active suicidal ideation were also excluded.

### Procedures

All participants involved in this study were enrolled in the Cohen Veteran Center Study and a Department of Defense funded project at New York University’s Langone Medical Center. As part of these studies, participants completed a battery of self-report assessments and the comprehensive neuropsychological battery WebNeuro. The self-report assessment included measures of depression, symptoms of PTSD, and trauma centrality as well as instruments not used in this particular work. Similarly, given our specific predictions of executive functioning, only the variables of cognitive flexibility and working memory were used from WebNeuro. In full, WebNeuro took participants roughly 40 min to complete and was administered with the participant alone in a windowless testing room.

### Measures

#### Post-traumatic Diagnostic Scale – Part III (PDS; Foa et al., 1997)

Part 3 of the Post-traumatic Diagnostic Scale (PDS) is a 17-item self-report questionnaire used to assess the severity of PTSD symptoms as related to a single event. Additionally, the PDS offers a diagnostic status for PTSD, which was used in this study to determine groups (α = 0.91).

#### Beck Depression Inventory-II (BDI-II; Beck et al., 1996)

The Beck Depression Inventory-II (BDI-II) is a 21-item self-report measure of depression that assesses the severity of various cognitive, behavioral, and physiological symptoms associated with depression (α = 0.93).

#### The Centrality of Event Scale (CES; Berntsen and Rubin, 2006)

The Centrality of Event Scale is a 20-item self-report measure that assesses how central an event is to a person’s self-identity and life story (e.g., this event was a turning point in my life) (α = 0.96).

#### Neuropsychological Assessment (WebNeuro, Brain Resource Company)

Subjects completed two test batteries via WebNeuro, an abbreviated form of IntegNeuro (Silverstein et al., 2007). Data from WebNeuro is linked to the standardized and integrative Brain Resource International database and cognitive functioning domain outputs were computed via WebNeuro software algorithms. For this study, norm-referenced sten scores were used for both variables in analyses.

#### Working Memory

Working memory was measured by the Digit Span task (forward and reverse), where the participant observed an increasingly growing sequence of numerical digits and was asked to recall the sequence correctly. The length of the longest correctly ordered sequence entered was recorded as the participant’s digit span.

#### Cognitive Flexibility

Cognitive flexibility was measured by Switching Attention task, a computerized adaption of the Trail Making test (Reitan, 1958). The participant was first presented with 25 numbers in circles and asked to click them with a mouse in an ascending numerical sequence with lines automatically drawn between clicked circles. The second part of the task consisted of the same basic task, but with a mixture of 13 numbers and 12 letters. The participant was instructed to switch back and forth between numbers in an ascending pattern (i.e., 1 A 2 B 3 C …). Performance for the Switching Attention Task was measure time taken to complete the task and accuracy.

## Results

SPSS version 21.0 (Statistics Package for Social Sciences, Inc. Chicago, IL, United States 2012) was used for all analyses. Group means and standard deviations for demographic characteristics and scores for the PDS, BDI, CES, and WebNeuro measures are presented in **Table 1**. Among the veterans exposed to traumatic events, there were significant differences between veterans with PTSD and veterans without PTSD in BDI, PDS, and CES scores. They did not significantly differ by age or in neurocognitive measures.

**Table 1:** Demographic and self-report data for OEF/OIF veterans.

	Veterans with PTSD		Veterans without PTSD		
Variable	*M*	*SD*	*M*	*SD*	*t*
**Demographic variable**					
Age	33.94	6.51	32.38	6.22	0.78
**Clinical and self-report measures**					
PDS	23.94	7.90	6.67	4.90	–7.99^∗∗^
BDI	16.12	9.82	4.08	4.51	–5.28^∗^
CES	68.00	18.95	54.08	22.15	–2.10^∗^
**Cognitive measures**					
Cognitive flexibility	5.59	1.88	5.38	1.94	–0.35
Working memory	5.18	2.42	4.48	2.45	–0.89

*OIF/OEF, Operation Enduring Freedom/Operation Iraqi Freedom; PDS, Post-traumatic Diagnostic Scale; BDI, Beck Depression Inventory; CES, Centrality of Events Scale; Cognitive measures assessed via WebNeuro, presented as sten scores.*

*^∗^*p* < 0.05; ^∗∗^*p* < 0.001.*

Bivariate correlations (**Table 2**) revealed significant (*p* < 0.05) relationships between the CES total and BDI total (*r* = 0.39), PDS current total (*r* = 0.53), cognitive flexibility scores (*r* = -0.33), and working memory scores (*r* = -0.36). The PDS total and BDI total were significantly correlated with each other (*r* = 0.85). Cognitive flexibility scores were significantly correlated with working memory scores (*r* = 0.54) as well as age (*r* = -0.38). Age was not found to significantly correlate with any additional measures.

**Table 2:** Correlations among clinical, self-report, and cognitive measures.

Variable	PDS	BDI	CES	Cognitive flexibility	Working memory
PDS		0.85^∗∗^	0.53^∗∗^	–0.16	–0.09
BDI			0.41^∗∗^	–0.00	–0.13
CES				–0.33^∗^	–0.36^∗^
Cognitive flexibility					0.54^∗∗^

*OIF/OEF, Operation Enduring Freedom/Operation Iraqi Freedom; PDS, Post-traumatic Diagnostic Scale; BDI, Beck Depression Inventory; CES, Centrality of Events Scale; Cognitive Flexibility and Working Memory assessed via WebNeuro.*

*^∗^*p* < 0.05; ^∗∗^*p* < 0.01.*

Two linear regressions assessing the effects of cognitive and clinical measures on centrality were then implemented. The dependent variable, CES total scores, was found to be normally distributed (K-S = 0.130, *p* = 0.079) without significant issues of kurtosis (-1.01, *SE* = 0.724) or skewness (-0.413, *SE* = 0.369). BDI and PDS scores were centered to account for multicollinearity (all transformed VIF values were below 4.09). The first regression (*F*_3,37_= 6.36, *p* < 0.05; *R*^2^= 0.34) found that CES total scores was *not* significantly predicted by cognitive flexibility but was approaching significance (*t* = -1.72, *p* = 0.09) when controlling for PDS total scores (*t* = 1.93, *p* = 0.06) and BDI-II total scores (*t* = -0.14, *p* = 0.89). In this model, there were no significant coefficients (*p* > 0.05). A subsequent model dropping BDI total scores (*F*_2,38_ = 9.79, *p* < 0.001; *R*^2^= 0.34) found that PDS was a significant predictor of CES scores (*t* = 3.67, *p* < 0.05) but cognitive flexibility was still only approaching significance (*t* = -1.83, *p* = 0.07).

The second linear regression assessing the effects of working memory on centrality (*F*_3,36_= 7.09, *p* < 0.05; *R*^2^= 0.37) found that working memory significantly predicted CES total score (*t =* -2.45, *p* < 0.05) when controlling for the PDS total scores (*t* = 2.63, *p <* 0.05) and BDI-II total scores (*t* = 0.84, *p* > 0.05). A subsequent linear regression using only the significant predictors of working memory and PDS total scores revealed similar results (*F*_2,37_ = 10.36, *p* < 0.01; *R*^2^= 0.36), with both working memory (*t* = -2.39, *p* < 0.05) and PDS (*t* = 3.65, *p* < 0.05) as significant coefficients.

## Discussion

This study establishes a link between neuropsychological functioning and trauma centrality. In addition to replicating a growing body of studies showing a positive correlation between trauma centrality and PTSD symptom severity, these findings show that lower levels of working memory and, albeit only approaching significance, cognitive flexibility are associated with trauma centrality when controlling for measures of depression and PTSD severity. This work highlights the need to consider how changes in neuropsychological processes may be linked to self-appraisals in the wake of traumatic events.

This work is novel in that it is the first to directly assess the relationship between trauma centrality and neuropsychological processes. Our findings are in line with the well-established phenomenon of overgeneralized autobiographical memory in PTSD. That is, the inability to successfully retrieve specific autobiographical memories has been linked to related deficits in working memory and executive control (e.g., Moore, 2009; Sumner, 2012). Furthermore, cognitive models of PTSD have proposed that executive functions, such as working memory, may underlie one’s ability to process and maintain adaptive self-representations after a traumatic event (e.g., Dalgleish, 2004). Deficits to working memory may then be the cause, or a reflection of, the inability to maintain and adapt self-representations, thus being tied to a person’s inability to see themselves and their autobiography outside of the context of their traumatic event. Future work would benefit from examining the extent to which neuropsychological deficits are associated with autobiographical memory and self-appraisals associated with trauma centrality.

The present study cannot determine whether it is neuropsychological deficits affecting trauma centrality, or vice versa, though previous research supports multiple explanations. Cognitive, clinical, and brain imaging studies have shown that self-related processes are supported by working memory and executive functioning, and damage to brain regions that support these processes often lead to profound alterations in self-identity (Kelley et al., 2002; Spreng et al., 2009). These studies suggest that deficits to working memory and executive functioning caused by trauma could therefore affect identity and trauma centrality. Alternatively, it is possible that impairments to self-related processes affect cognitive performance, where the working-self is biasedly selecting autobiographical recollections (present in the autobiographical knowledge base) that protect the self, even when those self-views may be maladaptive (Conway, 2005; Conway et al., 2005). While the direction of this relationship remains unknown, our findings are an important step to understanding how working memory and cognitive flexibility contribute to, or are affected by, autobiographical memory and identity following traumatic events.

Although our findings are promising, this work is limited in a number of ways. Principally, the direction of this relationship must be examined to determine whether it is centrality affecting neuropsychological functioning or vice versa, which these data cannot support due to the cross-sectional study design. In addition, although this study points to a link between neuropsychological functioning and trauma centrality, future work should aim to assess if lower neuropsychological functioning is a predisposition to both PTSD and centrality, or if PTSD is the source of neuropsychological decline. Additional measures and methods of testing working memory, cognitive flexibility, and executive functioning should also be employed to further determine the extent of the relationship between neuropsychological functioning and trauma centrality. For this study, a brief online assessment was used; however, in-person and more thorough assessment could provide additional insight into the complexity of working memory, cognitive flexibility, and neuropsychological functioning in general. An assessment of self-awareness would also be useful to determine any effects on self-administered measures (CES, PDS, BDI). In addition, those with PTSD may have had trouble attending to the on-line tasks. Thus future work would benefit from exploring these links with neuropsychological assessments that can better control for this potential artifact. Finally, this study’s generalizability is limited in that it only included male combat veterans. Future work should explore the relationship between centrality and neuropsychological functioning among diverse populations with varying traumatic events.

Despite these limitations, these findings begin to shed light on the potential mechanisms underlying trauma centrality, a construct strongly associated with PTSD. Such findings provide initial support for the possibility to the extent to which targeting working memory and cognitive flexibility in treatment may also help to promote self-representations that are less dominated by a traumatic event(s).

## Ethics Statement

This study was carried out in accordance with the recommendations of the Institutional Review Board (IRB) at the New York University (NYU) Langone School of Medicine with written informed consent from all subjects. All subjects gave written informed consent in accordance with the Declaration of Helsinki. The protocol was approved by the NYU Langone IRB.

## Author Contributions

RH, RB, NR, RAB, CM, and AB all contributed to study design, data collection, data analysis, and manuscript preparation. RT-P contributed to data analysis and manuscript preparation.

## Conflict of Interest Statement

The authors declare that the research was conducted in the absence of any commercial or financial relationships that could be construed as a potential conflict of interest.
